# Interferon Induction by RNA Viruses and Antagonism by Viral Pathogens

**DOI:** 10.3390/v6124999

**Published:** 2014-12-12

**Authors:** Yuchen Nan, Guoxin Nan, Yan-Jin Zhang

**Affiliations:** 1 Molecular Virology Laboratory, VA-MD College of Veterinary Medicine and Maryland Pathogen Research Institute, University of Maryland, College Park, MD 20742, USA; E-Mail: nyc@umd.edu; 2 Stem Cell Biology and Therapy Laboratory and Key Laboratory of Child Development and Disorders; Key Laboratory of Pediatrics in Chongqing, Chongqing International Science and Technology Cooperation Center for Child Development and Disorders, Children’s Hospital of Chongqing Medical University; Chongqing 400014, China

**Keywords:** interferon induction, TLR, RLR, viral antagonism

## Abstract

Interferons are a group of small proteins that play key roles in host antiviral innate immunity. Their induction mainly relies on host pattern recognition receptors (PRR). Host PRR for RNA viruses include Toll-like receptors (TLR) and retinoic acid-inducible gene I (RIG-I) like receptors (RLR). Activation of both TLR and RLR pathways can eventually lead to the secretion of type I IFNs, which can modulate both innate and adaptive immune responses against viral pathogens. Because of the important roles of interferons, viruses have evolved multiple strategies to evade host TLR and RLR mediated signaling. This review focuses on the mechanisms of interferon induction and antagonism of the antiviral strategy by RNA viruses.

## 1. Introduction

Host immunity can be divided into two broad categories: innate and adaptive. Innate immunity is known as non-specific resistance against infection, while adaptive immunity indicates acquired immune responses that are specific against invading pathogens. The innate immunity consists of physical, cellular and chemical components. The physical barrier includes skin and mucous membrane. The macrophages and natural killer cells are first line defenders against infection. Interferons are a major player in the chemical components of innate immunity against viral infection. Since the discovery of interferon in the 1950s, numerous studies have been conducted on members of this protein family. Along with the development of molecular biology, many genes encoding the interferons have been cloned and sequenced, as well as expressed in prokaryotic system. With the availability of purified interferons, interferon-based therapy has been developed and applied to treat cancer and viral infection. In this review, interferons, their classification and functions, induction pathways leading to their production, and virus-mediated antagonism will be described.

## 2. Interferons and Their Classification

Interferons are a large family of proteins that are genetically and functionally related [1]. The discovery of these mediators occurred in the 1950s. It was observed that virus-infected cells were resistant to secondary viral infection under certain circumstances. Similarly, it was also observed that treatment of cells with inactivated virus made cells resistant to infection by the same but live virus. Later, the term “interferon (IFN)” was coined to describe a special substance, possibly produced by cells, which could interfere with virus replication. However, due to technical limitations, it took more than two decades until interferons could be purified for further analysis [2,3]. The antigenic differences between IFNs generated by human fibroblasts and leukocytes led to the idea that IFNs constitute a protein family [4]. Currently, the functions of interferons are well-known, including antiviral activity, anti-proliferative activity, stimulation of cytotoxic T cells and modulation of the immune response [3]. Since their approval, purified interferons have also been used for the treatment of a variety of cancers and viral diseases [3].

Interferons are divided into three different types: type I, type II and type III (Table 1). In humans, type I interferons are the largest IFN family, which includes IFN-α, IFN-β, IFN-ε, IFN-κ and IFN-ω [5,6]. They belong to the class II family of α-helical cytokines, which includes type II IFN-γ, the newly identified type III IFNs, IL-10 and several IL-10 homologs [7,8,9]. Type I IFNs are encoded by individual genes except IFN-α, which has 13 subtypes [10]. IFN-δ, IFN-τ and IFN-ζ (or limitin) have also been identified as type I IFNs in swine, ruminants and mice, respectively [10]. Almost all cell types are capable of producing IFN-α/β; however, plasmacytoid dendritic cells (pDC) are considered as the major source for IFN-α production during the course of an infection [11,12].

All type I IFNs binds a ubiquitously expressed heterodimeric receptor complex, which includes the IFN-α receptor 1 (IFNAR1) and IFNAR2 as subunits. IFNAR2 serves for ligand binding, but both subunits are required to activate downstream signaling [13]. However, the various subtypes of type I IFNs display different binding affinities for the receptor complex and, thus, lead to various outcomes with respect to their antiviral, antiproliferative and immunomodulatory activity [14,15,16].

**Table 1:** The interferons and their receptors.

Type	Subtypes	Receptors
Type I	IFN-α (13subtypes)	IFNAR1 and IFNAR2
	IFN-β, IFN-ε, IFN-κ and IFN-ω	
	IFN-δ (swine), IFN-τ (ruminant) and IFN-ζ (mice)	
Type II	IFN-γ	IFNGR1 and IFNGR2
Type III	IFN-λ (1-4)	IFRL1 and IL-10R2

The type II interferon includes only IFN-γ, which functions as a homodimer [10]. Unlike type I IFNs, IFN-γ production is restricted to activated T cells, natural killer cells and macrophages [17]. Signaling for IFN-γ is transduced via the IFN-γ receptor complex (IFNGR). IFN-γ homodimer intimately interacts with two IFNGR1 subunits, with further binding of two IFNGR2 subunits, which results in receptor activation [18]. IFNGR is ubiquitously expressed, which means nearly all cell types are capable of responding to IFN-γ [17]. IFN-γ plays a major role in establishing cellular immunity; however, it is also able to induce expression of a group of genes that respond to type I IFN treatment [19,20].

The type III interferons are a newly discovered family of interferons, which comprise IFN-λ1, IFN-λ2, IFN-λ3 (also known as interleukin (IL)-29, IL-28A, and IL-28B, respectively) [10]. They were initially named “interferon-like cytokines” because IFN-λ is functionally similar to type I IFNs but has a distinct gene sequence and chromosomal location [13]. IFN-λ signals through a heterodimeric receptor composed of IFRL1 (or IL-28R) and IL-10R2, which are different from type I IFN receptors [5,7,10]. However, IFN-λ activates the same pathway and similar gene expression as type I IFNs. Recently, IFN-λ4 was described as the fourth member of the type III IFNs [21].

After the discovery of IFNs and their antiviral activity, viral RNA was proposed to be the potential inducer. Many early studies focused on the identification of possible nucleic acid inducers and numerous synthetic and biological RNAs were tested for this purpose. Unlike ssRNA, DNA or RNA:DNA hybrids, the dsRNA was found to be a potent trigger of the interferon response [22]. Moreover, dsRNA generated from DNA virus as well as other microorganism such as bacteria or artificially synthesized polyinosinic:polycytidylic acid (polyIC) were shown to be potent activators of IFN production [22]. However, a deep understanding of the mechanisms about how the host detects virus infection and initiates interferon synthesis did not occur until the recent years.

The discovery of Toll and the Toll-dorsal pathway in *Drosophila* led us to understand that there are conserved signaling pathways across the plants and animals as a common defense against pathogens [23]. Identification of the first Toll homologue in humans finally led us to set the concept of Toll-like receptors (TLRs) [24]. The TLRs were subsequently shown to recognize unique molecules present in a variety of pathogens from bacteria, fungi to viruses [25]. Now, we understand that pathogens are recognized by a group of pattern-recognition receptors (PRRs). Besides membrane-associated TLRs, there are another group of PRRs called RIG-I-like receptors (RLR), which comprise the cytoplasmic sensors of viral nucleic acids [26]. Host PRRs for RNA viruses include the RLR and TLR pathways. Both RLR and TLR3 can recognize double-stranded RNA (dsRNA) of the viral genome or replication intermediate of RNA viruses. Activation of RLR and TLR3 signaling leads to the activation of interferon regulatory factor-3 (IRF-3), IRF-7 and NF-κB (nuclear factor kappa-light-chain-enhancer of activated B cells). These transcription factors translocate to the nucleus and result in the induction of type I IFNs and expression of inflammatory cytokines. In the following sections, Toll-like receptors, RIG-I-like receptors and their corresponding signaling pathways are discussed further.

## 3. Toll-Like Receptor Pathway

### 3.1. Discovery of Toll in *Drosophila*

Toll, a gene in *Drosophila,* was identified during a study of genes essential for the establishment of the dorsal-ventral axis during embryo development [27]. Toll contains an extracellular domain, which consists of leucine-rich repeats (LRRs), flanked by cysteine-rich regions [28]. LRRs are found on a wide variety of proteins involved in some form of protein-protein interaction, such as receptors and adhesion molecules [29]. The cytoplasmic domain of Toll shows high similarity to the cytoplasmic domain of the mammalian interleukin-1 receptor (IL-1R) [30,31].

In 1994, the ligand of Toll in *Drosophila*, a novel secreted protein encoded by the *spätzle* gene, was identified and confirmed to be necessary to establish the dorsal-ventral pattern of the *Drosophila* embryo [32]. However, a later study demonstrated an additional, non-developmental role played by Toll that the activation of Toll could result in an immune response in *Drosophila* cell lines. This kind of immune response is mediated by two transcript factors from the *Rel* family, Dorsal-related immunity factor (Dif) and Dorsal, both of which are NF-κB like transcript factors in *Drosophila* [33]. Conclusive evidence from *in vivo* experiments demonstrated that Toll mediates signaling pathways and the extracellular Toll ligand, *spätzle*, controls the expression of the antifungal peptide gene drosomycin in *Drosophila* adults [34]. Mutations in the Toll signaling pathway dramatically reduce the survival of *Drosophila* after fungal infection [34]. These discoveries led to the identification of a conserved signaling pathway of the immune response: the *Drosophila* Toll-Dorsal pathway, which is considered the homologue of the interleukin-1 receptor (IL-1R)-NF-κB pathway in mammals [23].

### 3.2. Discovery of Toll-Like Receptors in Mammalian Hosts

After revealing the role played by Toll in the immunity of *Drosophila*, researchers started to look for the analogous genes in humans that are involved in this evolutionally conserved immune signaling pathway. However, the idea for the existence of certain cellular receptors that could sense pathogens and deliver signals to cells was spawned even earlier, in 1989 [35]. In 1997, the first human homologue of *Drosophila* Toll, later known as TLR4, was cloned and confirmed to induce the activation of NF-κB and the expression of NF-κB activated inflammatory cytokines [24]. However, since little knowledge about the existence of the natural ligand or the human homologue of the *spätzle* gene was available for the first discovered Toll homologue in humans, the strategy employed to confirm its function relied on artificial modification of this Toll homologue. Based on the previous data for generating the constitutively active mutant of *Drosophila* Toll, a similar mutant construct was made and fused with CD4 ectodomain as a marker to verify the expression of the hybrid protein. When this CD4–Toll fusion protein was overexpressed in a macrophage-derived cell line, it constitutively activated NF-κB and resulted in the upregulation of inflammatory cytokines as well as costimulatory molecules, as originally predicted [24]. Later, a total five Toll homologues from human were identified, and designated as TLR1 to 5 [36].

The term TLR was initially assigned to describe a Toll related gene that contributes to hedgehog function in the adult eye of *Drosophila* in 1994 [37]. Usage of this term occurred earlier than the discovery of Toll as an immune response related receptor. However, since the discovery of Toll homologues in mammalian hosts, TLR has been assigned to describe a new receptor family more closely related to *Drosophila* Toll homologs rather than to vertebrate IL-1Rs [36].

Currently, a total of 13 TLRs have been identified in humans and mice (TLR1-13) [38,39,40,41,42,43]. TLR1 to TLR10 are expressed in humans, while TLR11 to TLR13 are found exclusively in the mouse. There is indeed a TLR10 homologue identified in the mouse; however, a retroviral insertion disrupted the *TLR10* gene and rendered it non-functional [44]. Furthermore, increasing evidence shows that other mammals may express TLRs that are not found in humans. Some TLRs, such as TLR14, TLR5S, TLR20 and TLR21 have been identified in Takifugu pufferfish and catfish, and may be found in non-mammalian species with unique properties distinct from those in mammals [45,46,47]. Of the human TLRs, TLR3, TLR7, TLR8 and TLR9 are membrane proteins localized in the endosome while the remaining TLRs are mainly located on the cell surface [48].

### 3.3. TLR Ligands

After the discovery of TLRs in humans, a key question remained unanswered: Do human TLRs recognize natural ligands from pathogens or are there human homologues of the *spätzle* gene that serve as ligands for TLRs [49]? Initial data from HEK293 cells indicated that TLR2 mediates LPS-induced signaling when co-expressed with CD14 [50]. However, the conclusive answer came unexpectedly from a study in a mutated TLR4 gene in the LPS-unresponsive C3H/HeJ mouse strain [51]. Before the identification of TLR in mammals, researchers were unable to identify the gene responsible for the LPS mediated response in different mouse strains with different phonotypes, and they termed this proposed gene *LPS* [52,53,54,55]. In 1998, a mutation in the TIR domain of TLR4 was finally identified and led to defective LPS signaling in C3H/HeJ and C57BL/10ScCr mice, which confirmed that LPS is the ligand for TLR4 [51]. Later, TLR4 was confirmed to be the product of the proposed *LPS* gene [56]. Furthermore, other studies indicated that another molecule, MD-2, which non-covalently binds to the extracellular domain of TLR4, serves as the real “receptor” for LPS, whereas TLR4 serves as the signal-transducing component of the larger “TLR4 signaling complex” [57]. Since then, more TLR ligands have been identified.

TLR1 in association with TLR2 as a heterodimer recognizes peptidoglycan and lipoproteins [58,59,60]. The dsRNA is recognized by TLR3 [61]. Bacterial flagellin is recognized by TLR5 [62]. TLR6 recognizes lipopeptide cooperatively with TLR2 [58]. TLR7 mainly recognizes single-stranded RNA, a common feature of most RNA viral genomes [63]. Some artificially synthesized compounds, such as imiquimod, can be recognized by TLR7 as well [64]. TLR7 and TLR8 are functionally related, as TLR8 recognizes viral RNA and self-RNA within snRNP autoantibody complexes [65]. Unmethylated CpG DNA derived from bacteria is recognized by TLR9 [66]. Currently, no specific ligand had been identified for TLR10. It has been shown that TLR10 plays a role in mediating bacterial peptidoglycan-induced trophoblast apoptosis, which implies that TLR10 may recognize a bacteria-derived component [67].

In summary, within the TLR family, TLR3, 7, 8, and 9 represent a subfamily that are localized in intracellular compartments, such as endosomes and the endoplasmic reticulum (ER), where they recognize viral nucleic acid species [48]. There is cell-specific expression of those four TLRs. Innate immune cells express the endosomal TLR 7, 8 and 9, which sense GU-rich RNA and CpG-containing DNA, while TLR3 is expressed in broad cell types, such as endothelial cells, fibroblasts and astrocytes [68]. On the other hand, the other subfamily includes TLR1, 2, 4, 5, and 6, which are localized on the cell surface and mainly recognize bacterial cell wall components [48].

Recently, the connection between autoimmune disease and TLRs activated by endogenous ligands was revealed [69]. There is increasing evidence indicating that some endogenous ligands can activate TLR7 and TLR8 and result in autoimmune disease in mouse models [70]. TLR8 and 9 are believed to be involved in the development of some autoimmune diseases such as rheumatoid arthritis. Endogenous DNA fragments generated during cell death, either apoptosis or necrosis, are believed to serve as the endogenous ligand for these TLRs [69,71]. A full list of TLR ligands is listed in Table 2.

**Table 2:** Toll like receptors and their ligands.

TLRs	Adaptor(s)	Ligand [Ref]
TLR1	MyD88	Peptidoglycan/lipoproteins [58,59]
TLR2	MyD88	Peptidoglycan/lipoproteins [58,59,60]
TLR3	TRIF	dsRNA [61]
TLR4	MyD88/TRIF/TRAM/TICAM	LPS [56]
TLR5	MyD88	Bacterial flagellin [62]
TLR6	MyD88	Lipopeptide [58]
TLR7	MyD88	ssRNA [63,64]
TLR8	MyD88	ssRNA [65]
TLR9	MyD88	CpG DNA [66]
TLR10	MyD88	Unknown
TLR11	MyD88	Unknown
TLR12	MyD88	Unknown
TLR13	MyD88	Unknown

### 3.4. TLR Signaling

**Figure 1:**
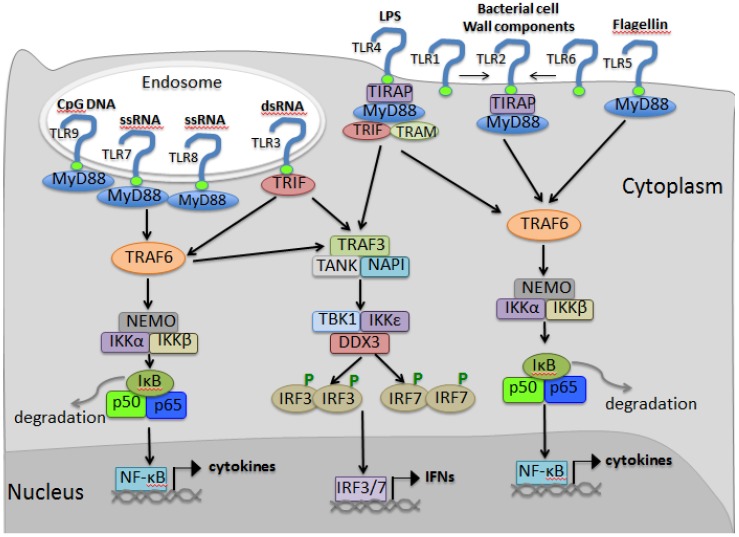
Schematic illustration of toll-like receptors (TLR)-mediated signaling. Cytoplasm and nucleus are indicated separately. TLRs are indicated on cell membrane or in endosomes according to their distribution. Their ligands and adaptors are shown. Activation of the TLRs by ligand binding leads to downstream signaling, resulting in production of type I interferons (IFNs) and inflammatory cytokines. Additionally, note that transcription activators NF-κB (nuclear factor kappa-light-chain-enhancer of activated B cells) and AP-1 (the activator protein 1) also contribute to the IFN production; but for clarity, they are not shown in this illustration.

TLRs are believed to function as dimers. Schematic illustration of the TLR signaling is shown in Figure 1. After recognition of their ligands, TLRs activate the same signaling components as those used in IL-1 receptor (IL-1R) signaling due to a conserved Toll-IL-1R (TIR) domain in intracellular regions [72]. As a result, TLRs recruit a set of adaptor proteins with TIR domains by homophilic interaction of their TIR domains. Those adaptor molecules include myeloid differentiation primary response gene 88 (MyD88), TIR-containing adaptor protein/MyD88-adaptor-like (TIRAP/MAL), TIR-containing adaptor inducing interferon-β/TIR-domain-containing adaptor molecule 1 (TRIF/TICAM1) and TIR-domain-containing adaptor molecule/TRIF-related adaptor molecule 2 (TICAM2/TRAM) [73]. Adaptor molecules used by TLRs are varied for different TLRs. TLR4 uses all four adaptors while TLR3 uses only TRIF [73].

The most well-known adaptor molecule for TLRs is MyD88, which is utilized by all TLRs except TLR3. MyD88 is composed of a death domain as well as a TIR domain. Upon TLR activation, a homophilic interaction occurs between the death domain of MyD88 and members of the IRAK (IL-1 receptor-associated kinase) family, including IRAK1 and IRAK4 [74,75]. IRAK4 is initially activated, which in turn phosphorylates and activates IRAK1. Then, phosphorylated IRAK4 and IRAK1 dissociate from MyD88 and interact with TRAF6, which is a RING-domain E3 ubiquitin ligase. TRAF6 promotes the Lys63-linked polyubiquitination of itself and NEMO (NF-κB essential modulator, or IKKγ) [76]. Ubiquitinated NEMO and TRAF6 subsequently recruit a protein kinase complex involving transforming growth factor-β activated kinase 1(TAK1) and TAK1 binding proteins (TABs) [77]. TAK1 and the TABs (TAB1 and TAB2) then activate two distinct pathways involving the IKK complex and MAPK. TAK1 promotes downstream activation of the IκB kinases (IKK): IKKα and IKKβ. These IKKs directly phosphorylate the inhibitory IκB family members, which normally sequester NF-κB in its inactive form in the cytosol. However, phosphorylation of IκBs leads to their polyubiquitination and degradation, followed by the activation and nuclear translocation of NF-κB. The activation of the canonical NF-κB pathway results in the induction of inflammatory cytokines and co-stimulatory molecules [72].

A notable function of MyD88 is that signaling transduced via MyD88 due to activation of TLR7 and TLR8 in plasmacytoid dendritic cells (pDCs) results in induction of IFNs [78]. TLR7 and TLR9, are selectively expressed by pDCs (also known as professional IFN-producing cells), which are a subset of DCs with a plasmacytoid morphology and unique in their capacity to rapidly secrete type I IFNs in response to viral infection [12,79]. In pDC, IRF-7 is constitutively expressed and binds MyD88 to form a signaling complex with IRAK4, TRAF6, TRAF3, IRAK1 and IKKα [80,81]. Within this complex, IRF-7 becomes phosphorylated, dissociates from the complex, and translocates into the nucleus to activate IFN induction upon activation of TLR7 and TLR8.

Besides MyD88, TRIF is another adaptor molecule that is specially utilized by TLR3 and represents another important pathway for TLR signaling [73]. Upon activation of TLR3 by dsRNA, TRIF interacts directly with the TIR domain of TLR3 [82]. TRIF also directly binds TRAF6 via its TRAF6-binding motifs in the N-terminal region [83]. TRAF6 then activates TAK1 in a manner similar to that of the MyD88-dependent pathway for NF-κB activation. On the other hand, TRIF has been confirmed to associate with both IKKε and TBK1 via its N-terminal region, which shares binding with TRAF6; the TANK family protein NAK-associated protein 1 (NAP1) might facilitate the interaction of both kinases with TRIF [83,84]. TBK1 and IKKε possess essential roles in the induction of type I IFNs through phosphorylation and activation IRF-3 and IRF-7 [85]. Upon activation, IRF-3 forms a homodimer, which translocates to the nucleus and binds to its target sequences, such as the promoter region of the IFN-β gene. Thus, there is a TLR3-TRIF-IKKε (TBK1)-IRF-3 signaling axis which finally leads to IFN production.

TIRAP and TRAM are another two adaptors that are less investigated, but still play important roles in TLR signaling. Their functions are related to TLR4-mediated signaling. TLR4 is the only TLR that uses all four types of the adaptors. With assistance from TRAM, TLR4 can function via TRIF as well to activate TRIF-IKKε (TBK1)-IRF-3 signaling axis. TRAM, also known as TICAM-2, acts as a bridging adaptor between TLR4 and TRIF [86]. TIRAP has a crucial role in the MyD88-dependent signaling pathway shared by TLR2 and TLR4. Just like TRAM, TIRAP serves as a “bridge” for MyD88 and receptors and thus activates NF-κB [87,88].

Taken together, as the consequence of activation, all TLRs commonly promote chemokine and pro-inflammatory cytokine expression via NF-κB. However, the hallmark of endosomal TLR3, 7, 8 and 9, which sense nucleic acids, is the induction of type I IFNs [48].

## 4. RIG-I Like Receptor Pathway

### 4.1. Discovery of the RIG-I Like Receptor

Retinoic acid-inducible gene I, or RIG-I (also known as DDX58), is a member of the so-called DExD/H box RNA helicase family. DExD/H box RNA helicase and its related DEAD box helicase, named according to one of the conserved protein motifs, belong to helicase superfamily 2 [89,90]. This is the largest helicase group and, in fact, a subset of it includes proteins from *E. coli*, eukaryotes, even from DNA and RNA virus [89,90]. RIG-I-like receptors (RLRs) currently include three members: RIG-I, melanoma differentiation-associated gene 5 (MDA5, also known as Helicard or IFIH1) and Laboratory of Genetics and Physiology 2 (LGP2) [91]. Unlike membrane-associated TLRs that detect pathogen-associated molecular patterns (PAMPs) derived from viruses, bacteria and fungi, RLRs are cytoplasmic proteins that are specific for detecting RNA derived from viruses in the cytosol [68].

RIG-I was originally identified as a gene induced by retinoic acid during the differentiation of an acute promyelocytic leukemia cell line [92,93]. In 1997, a Chinese research group from the Shanghai Institute of Hematology, which focused on employing all-trans retinoic acid (ATRA) as a treatment for acute promyelocytic leukemia, cloned a novel set of ATRA-inducible genes when using retinoic acid to induce the differentiation of an acute promyelocytic leukemia cell line. This group designated these genes as “RIG-A, RIG-B, RIG-C,” and so on. They deposited the sequence of the genes into GenBank, including the sequence for RIG-I [91,94]. Later, another study identified a RIG-I homologue in swine, and named it RNA helicase induced by virus (RHIV-1). This gene was upregulated when porcine pulmonary alveolar macrophages were infected with porcine reproductive and respiratory syndrome virus (PRRSV) [93]. Another group from Japan also reported that stimulation of endothelial cells with LPS led to the up-regulation of RIG-I [92]. Since then, more reports have indicated that RIG-I can be up-regulated in cells infected with a virus or cells treated with IFN-γ [95,96]. Taken together, the earlier reports connected RIG-I with innate immunity and IFNs.

The first report to describe the function of RIG-I came from Yoneyama *et al.* [97], who found that the helicase domain of RIG-I is responsible for dsRNA recognition and the caspase activation and recruitment domain (CARD) for transmitting the signal downstream, leading to the activation of NF-κB and IRF-3 and then IFN induction. After revealing the function of RIG-I, the same group started searching mammalian gene databases for RIG-I related genes, leading to the discovery of two other members of the RLR family, MDA5 and LGP2 [98].

MDA5 was identified in a differentiation induction subtraction hybridization (DISH) screen, which was designed to define genes regulated by the induction of terminal differentiation in human HO-1 melanoma cells [99,100]. The genes identified by the DISH screen were named melanoma differentiation-associated (MDA) genes. MDA5 was one of these novel genes that was induced by IFN-β in human melanoma cells and had melanoma growth-suppressive properties [101]. MDA5 shares 23% and 35% identical aa with RIG-I in the CARD and helicase domains, respectively [98].

LGP2, the latest member of the RLR family discovered, was identified based on a set of studies in mammary gland development and remodeling, which are regulated by two members of the STAT (signal transducer and activator of transcription) family, STAT3 and STAT5. Since *Stat3*, *Stat5a*, and *Stat5b* are closely related in a region of mouse chromosome 11, this led the research team to hypothesize that additional genes involved in mammary gland development might harbor in this locus [102,103]. Finally, two novel genes, *Lgp1* and *Lgp2*, were identified and LGP2 was found as a cytoplasmic protein harboring a DExH/D-box helicase domain [103]. Unlike RIG-I and MDA5, which contain both the CARD and helicase domains, LGP2 contains only the helicase domain with 31% and 41% identical aa residues to the helicase domains of RIG-I and MDA5, respectively [98].

### 4.2. Ligands of RLRs

RLRs present in the cytosol of all cell types to induce type I IFNs and cytokines upon activation [68]. As RNA helicases, RLRs mainly recognize RNA molecules derived from viruses, and in some cases, RNA from bacteria [68]. However, ligand preference varies among different RLRs. In particular, even though the details of the RLRs ligands have been revealed, there are still many questions remaining. Currently, RIG-I is better defined than MDA5 and LGP2, and considerable efforts have been made to identify its ligands.

Initially, polyIC was used as an artificial ligand for RIG-I to activate IFN production, consistent with a previous concept of dsRNA as a physiological viral trigger for IFN induction due to the simple explanation that RNA viruses are bound to make mistakes during replication and are therefore likely to expose at least some dsRNA molecules in the cells [22]. However, this assumption was questionable. Studies employing dsRNA-specific antibodies have shown that positive-stranded RNA and DNA viruses make dsRNA during replication; however, negative-strand RNA viruses do not appear to produce detectible amounts of dsRNA, such as influenza A virus [104]. On the other hand, an important addition to this field was the discovery that a 5′ triphosphate (5′ppp) group on an RNA molecule serves as a activator of RIG-I in addition to dsRNA [105,106]. This discovery gives a potential explanation to the earlier observation that 5′ triphosphate bearing siRNA generated from *in vitro* transcription is able to induce interferon induction, while removing the 5′ triphosphate from artificially synthesized siRNA maintains full efficacy but no longer induces IFNs [107].

On the other hand, later studies challenged the earlier observation that the 5′ triphosphate alone is sufficient for RIG-I activation and indicated the requirement for a double-stranded component in addition to the triphosphate [22]. Specifically, synthetic 5′ppp ssRNA was not capable of inducing IFNs when introduced into cells, while the same RNA molecule generated by *in vitro* transcription served as a competent activator of the IFN response since the RNA mixture from the T7 transcript contained a significant portion of dsRNA molecules [22]. Further characterization of RIG-I activation requirements showed that dsRNA complementarity of at least 10–18 nt is required at the 5′ppp containing end in order to induce RIG-I activity. These types of RNA molecules can form a panhandle structure, and is supported by the data that ends of defective interfering (DIs) genomes from negative-stranded RNA viruses are very potent IFN inducers [108]. Thus, for a negative-stranded RNA virus, the complementary 5′ and 3′ terminal sequences of its genomes bear the potential to hybridize as dsRNA panhandle structures with blunt ends and 5′ppp groups, thereby meeting the requirements of a RIG-I ligand [109].

In addition to 5′ triphosphate dsRNA, another study defined the RIG-I ligand as blunt-ended dsRNA longer than 23 bp with higher activity when the RNA contains two blunt ends [110]. Since the dsRNA antibody recognizes dsRNA longer than 40 bp, this ligand size requirement gives a potential explanation for why dsRNAs generated by negative-stranded RNA virus replication were not detected since they are associated with nucleocapsid proteins and exposed part may be too short for the antibody detection [68]. Moreover, a recent study indicated that RIG-I is also involved in DNA-initiated IFN induction [68]. dAdT functions as a template for endogenous RNA polymerase III, which generates 5′triphosphorylated AU-polymers in the cytosol to stimulate RIG-I [111]. A recent study showed that base-paired RNAs with 5′-diphosphates also serve as RIG-I agonists to induce IFNs [112].

There has been less attention on the ligand of MDA5. Although polyIC indeed binds and stimulates RIG-I, it is worth noting that polyIC fails to induce IFN-α when injected intravenously into MDA5-deficient mice or transfected *in vitro* into MDA5-deficient cells [113,114]. While RIG-I is essential for interferon induction for paramyxoviruses, influenza virus and Japanese encephalitis virus, MDA5 is critical for picornavirus detection [114]. This implies that MDA5 and RIG-I helicases have differential roles in the recognition of RNA viruses. Another study demonstrated that MDA5 was only stimulated by long polyIC fragments when using polyIC fragments of different sizes generated from RNase-III digestion as the activator [115]. Briefly, dsRNA of 1 kb was entirely dependent on RIG-I for IFN induction, while increasing the length to 4 kb progressively led to dual MDA5 and RIG-I dependence. MDA5 can recognize dsRNA more than 7 kb long. This also shows that viral dsRNAs differentially activate RIG-I and MDA5, depending on their length as well. Furthermore, an mRNA fragment from the negative-stranded RNA parainfluenza virus 5 activated type I IFN expression in a MDA5-dependent manner [116]. Since type I IFN induction by this mRNA fragment requires the involvement of RNase L, it implies that RNase L may recognize and process viral mRNA into a MDA5-activating structure [116].

As the third member of the RLRs, LGP2 is not fully understood and the current data on its function are controversial. No specific research has been performed on ligand identification for LGP2. Unlike RIG-I and MDA5, LGP2 structurally lacks CARD domain, suggesting a putative ligand sequestering role [68]. Indeed, earlier reports suggested an immune suppressive function for LGP2 [117,118,119]. Moreover, a mutant LGP2 with abolished RNA binding ability was still able to inhibit RIG-I mediated signaling [120]. However, further studies on LGP2-deficient mice revealed an indispensable role of LGP2 for the immune response to viruses that are mainly detected by MDA5 [121,122]. In some cases, an impaired RIG-I antiviral response was observed as well [121]. Another study also demonstrated that LGP2 contributed to sustained RLR signaling for IFN-β expression in myeloid cells, and that LGP2 promotes an essential pro-survival signal in response to antigen stimulation to induce CD8+ T cell expansion and effector functions against divergent RNA viruses [123]. Therefore, although no final conclusion has yet been made, LGP2 appears to have a modulatory role in fine-tuning the innate immune response to viruses depending on the context of viral infection.

Collectively, the current data indicate that RIG-I and MDA5 recognize different types of viruses with a possible overlapping recognition by both, while LGP2 plays a role as a potential regulator. However, both RIG-I and MDA5 require the same adaptor molecule, mitochondrial antiviral signaling (MAVS) (also known as virus-induced signaling adaptor (VISA), IFN-β promoter stimulator 1 (IPS-1) and the CARD adaptor inducing IFN-β (Cardif)) to activate innate immune defense. In the next section, the downstream signaling pathway of RLRs will be discussed.

### 4.3. Signaling of RLRs

**Figure 2:**
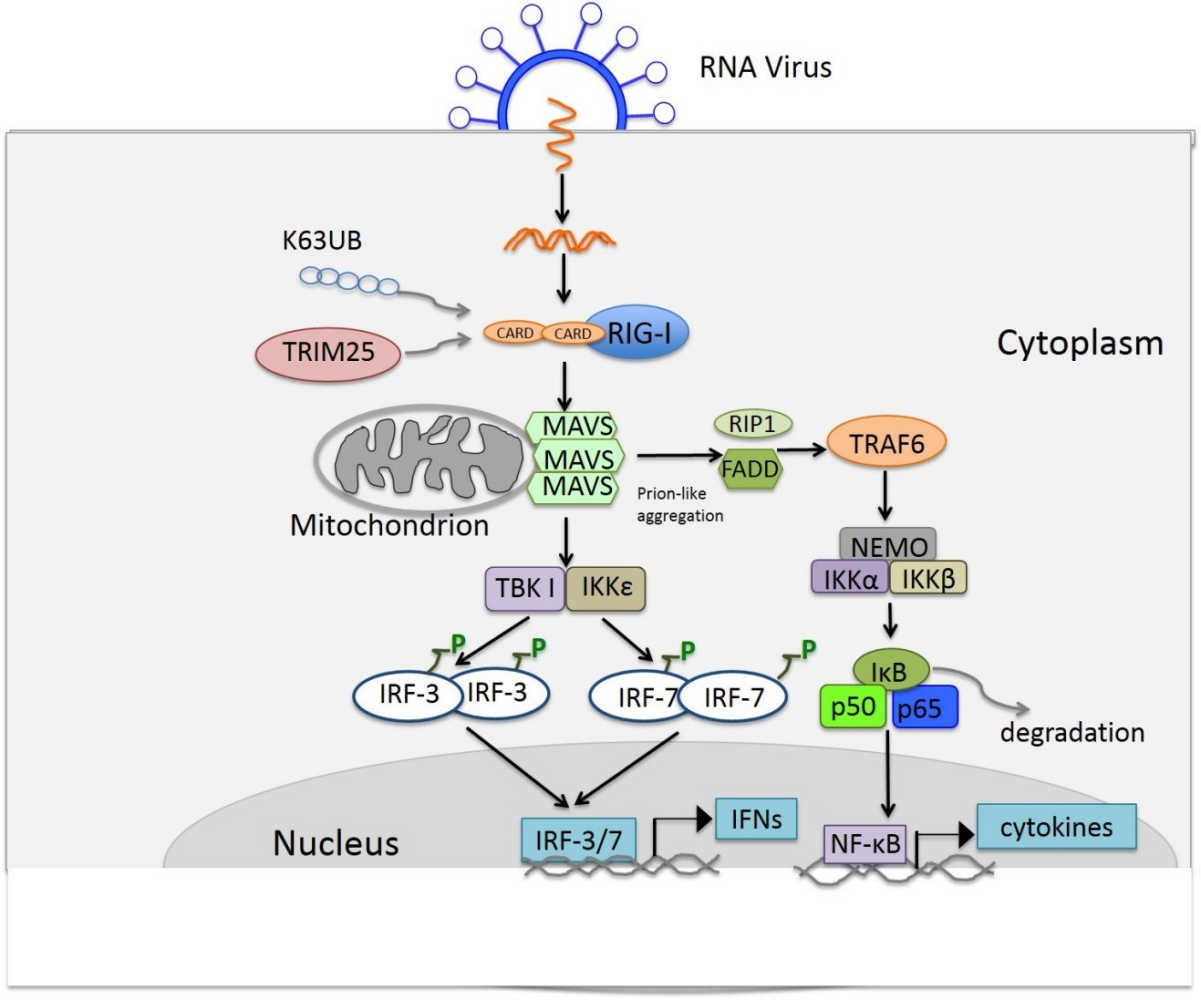
Schematic illustration of retinoic acid-inducible gene I (RIG-I) mediated signaling. A virion of RNA virus is indicated on cell surface and a double-stranded replication intermediate viral RNA is shown as the ligand for RIG-I. The ligand binding activate RIG-I, shown as lysine-63 (K63)-linked polyubiqutination by tripartite motif (TRIM)25, an E3 ubiquitin ligase. The activated RIG-I binds to adaptor mitochondrial antiviral signaling (MAVS), leading to its prion-like aggregation and activation of downstream signaling and consequent production of type I IFNs and inflammatory cytokines. Additionally, note that NF-κB and AP-1 also contribute to the IFN production; but for clarity, they are not shown in this illustration.

When RLRs were found to be the intracellular receptor of viral RNAs, it was less clear which molecule acts downstream of these helicases and facilitates the activation of IRF-3, IRF-7 and NF-κB, which are required for IFN transcription and utilized by TLRs. Soon thereafter, MAVS was identified as the necessary adaptor for RLR signaling from different research groups with different names (MAVS, IPS-1, VISA or Cardif. hereinafter, MAVS is used) assigned to the same molecule [124,125,126,127]. MAVS contains an N-terminal CARD-like domain just like RLRs and a C-terminal transmembrane domain which defines its mitochondrial localization. Both domains are essential for MAVS signaling [127]. MAVS also interacts with TRIF, TRAF6, IKKα, IKKβ and IKKε, thus leading to the activation of NF-κB and IRF-3 [125,126]. Although the details of these interactions and activation pathways are still incomplete, available data for RLR signaling indicates a schematic picture. Briefly, in the absence of its ligand, RIG-I is in an inactivated form. Binding of dsRNA or 5′ppp-RNA to the basic cleft in the C-terminal domain (CTD) induces a conformational change, which causes uncovering of the CARD domain in the presence of ATP [128]. TRIM25, a member of the tripartite motif (TRIM) protein family, is an E3 ubiquitin ligase reported to bind to the N-terminal CARD of RIG-I and conjugate the lysine 172 in the internal CARD with a lysine 63-linked polyubiquitin chain [129,130]. Then, the CARD domain of RIG-I interacts with the CARD domain of MAVS, which causes the conversion of MAVS on the mitochondrial membrane to a prion-like aggregate [131]. MAVS then activates the cytosolic kinases IKKs and TBK1, which activate the transcription factors NF-κB and IRF-3. Finally, type I interferons and cytokines are induced [132]. A schematic illustration of RIG-I mediated IFN induction is shown in Figure 2.

MAVS was also identified in peroxisomes as well [133]. Functional analysis indicates that peroxisomal and mitochondrial MAVS acting sequentially for their antiviral function. Peroxisomal MAVS mediated the rapid interferon-independent expression of defense factors to provide the short-term protection upon viral infection [133]. However, mitochondrial MAVS activates an interferon-dependent signaling pathway with delayed kinetics [133]. Furthermore, another study demonstrated peroxisomal MAVS could activate IRF-1 to induce type III IFNs [134]. These latest discoveries have enhanced our understanding of pathways leading to IFN induction.

On the other hand, in the RLR-MAVS-IRF-3/7 axis, another study identified that receptor-interacting serine-threonine kinase 1 (RIP1) and Fas-associated death domain (FADD) are involved. Both RIP1and TBK1 are required for the activation of the transcription factor IRF-3 [135]. Moreover, lysine-63 (K63) polyubiquitin chain-mediated ubiquitination of TRAF6 and TBK1 is also required for the activation of TRAF6 and TBK1 [136,137]. Furthermore, a recent study identified TRIM14 as a positive factor for RLR-mediated signaling pathway [138]. TRIM14 localizes at the outer membrane of mitochondria and interacts with MAVS. Upon viral infection, TRIM14 undergoes K63–linked polyubiquitination at Lys-365 and recruits NEMO to the MAVS complex, leading to the activation of downstream signaling [138].

## 5. Other Functions of TLRs and RLRs

TLRs and RLRs form the most important sensor families for viral infection, resulting in an innate immune response within minutes of infection to produce type I IFNs and pro-inflammatory cytokines. For TLRs, besides their important roles played in host innate immunity, TLR-mediated signaling is involved in many other biological processes. TLRs have also been shown to be an important linker between innate and adaptive immunity through their presence in dendritic cells (DCs) [139]. TLR activation in DCs causes an enhanced display of MHC peptide ligands for T cell recognition. TLR activation can also up-regulate co-stimulatory molecules that are important for T cell clonal expansion and the secretion of immunomodulatory cytokines to direct T cell differentiation into effectors [140]. TLRs are able to regulate neutrophil migration, activation and apoptosis [141]. TLR signals also regulate B-cell activation and survival [142]. Due to their unique feature linking innate and adaptive immunity via DCs, considerable effort has been made to testing TLR ligands as novel vaccine adjuvants to enhance the efficacy of vaccination.

TLRs also play important roles in some diseases and are regarded as potential therapeutic targets. It was demonstrated that TLR4 plays a crucial role in the development of contact allergy to nickel [143]. The application of a TLR4 antagonist in the treatment of severe sepsis entered into clinical trial as well [144]. Moreover, besides their roles in the immune system, TLRs in the intestinal epithelium affect intestinal function [145], and TLRs are also able to modulate adult hippocampal neurogenesis [146]. Activation of TLR3 is required for efficient nuclear reprogramming during the generation of induced pluripotent stem cells (iPSCs) [147].

For RLRs, other than its ability to initiate an innate response, RIG-I has been proposed to participate in a variety of intracellular events. ATRA-induced differentiation and normal myelopoiesis are accompanied by the induction of RIG-I [91,94]. Conversely, disruption of the RIG-I in mice leads to the development of a progressive myeloproliferative disorder [148]. It has been suggested that RLRs also participate in cell differentiation, and RIG-I participates in TLR-stimulated phagocytosis [94]. *In vivo* studies and work in clinical samples suggest that RIG-I may be involved in immune responses associated with both non-infectious and infectious diseases, such as atherosclerosis, psoriasis and rheumatoid arthritis [91].

Taken together, TLRs and RLRs play crucial roles both in immunity and non-immune events. As IFNs are the key consequence of TLR and RLR activation, IFNs induce the synthesis of proteins with antiviral activity.

## 6. Viral Antagonism of IFN Induction

### 6.1. Antagonism *via* Direct Interaction with Host Molecules in the IFN Induction Pathway

It is well known that viruses employ multiple strategies to evade host TLR and RLR mediated signaling. Some viral proteins can directly interact with host signaling molecules to inhibit their functions (Table 3). For example, the NS1 protein of influenza A virus is able to interact with RIG-I to block downstream signaling [149]. RIG-I, MAVS and NS1 of influenza A virus can form an insoluble complex in the cells. The V protein of paramyxoviruses binds to MDA5 and inhibits its downstream activation [150]. Respiratory syncytial virus (RSV) NS1 protein can associate with MAVS during early stage of the viral infection, and this association disrupts the RIG-I and MAVS interaction as well as downstream signaling [151]. The γ_1_34.5 protein of HSV forms a complex with TBK1 and disrupts the interaction of TBK1 and IRF-3, which prevents the induction of interferons [152]. HSV mutant lacking γ_1_34.5 cannot replicate efficiently in normal cell but replicate efficiently in TBK1(-/-) cells, indicating a key role for inhibiting IFN induction by γ_1_34.5 [152]. The Ebola virus protein VP35 interacts with IKKε and TBK1 to impair the activation of IRF-3 [153]. When overexpressing in cells, VP35 impairs IKKε-IRF-3, IKKε-IRF-7, and IKKε-IPS-1 interactions, suggesting that VP35 exerts its IFN-antagonist function by blocking necessary interactions between the kinases IKKε and TBK1 and their partners [153]. Moreover, Dengue virus NS2B/3 protease can interact with IKKε to inhibit IFN induction as well [154]. Human papillomavirus 16 (HPV16) E6 oncoprotein binds to IRF-3 and inhibits its transcriptional activity as well [155].

### 6.2. Antagonism *via* Degradation or Cleavage of Host Molecules in the IFN Induction Pathway

Viruses encode proteins that target host molecules for degradation or cleavage to inhibit the production of IFNs (Table 3). For example, viral-encoded 3C protease of encephalomyocarditis virus (EMCV), a member of picornavirus, induces RIG-I degradation during EMCV infection [156]. In the *in vitro* assay, bacterial expressed 3C protease was able to cleave RIG-I and generate a 49 kDa cleaved product in a dose dependent manner. Mutation of 3C protease catalytic core abolished RIG-I cleavage, indicating RIG-I acts as the substrate for the viral protease. Moreover, EMCV infection causes caspase-mediated degradation of RIG-I, which further inhibits host RIG-I signaling [156]. As another member of picornavirus, hepatitis A virus protease-polymerase processing intermediate 3CD can cleave TRIF to inhibit TLR3-mediated signaling [157]. Interestingly, TRIF can be only cleaved by intermediate 3CD of HAV, but not by the mature 3C protease or the 3ABC precursor, which can cause degradation of MAVS [157]. What is more, HAV 3CD-mediated TRIF degradation requires both the cysteine protease activity of 3C protease and downstream 3D polymerase sequence, but not 3D polymerase activity [157]. However, for the coxsackievirus B3 virus, which is also a member of picornavirus, its 3Cpro cysteine protease is able to function alone to cleave both MAVS and TRIF to evade host innate immune responses [158].

Besides RIG-I and TRIF, MDA5 is also targeted by viruses as well. During the poliovirus infection, MDA5 protein is degraded in poliovirus-infected cells [159]. However, cleavage of MDA5 during poliovirus infection is a proteasome and caspase dependent process and is not carried out by poliovirus encoded proteases 2Apro or 3Cpro [159]. Moreover, as the key factor for IFN induction, IRF-3 is targeted by Npro of Classical swine fever virus (CSFV) for proteasomal degradation [160]. Inhibition of the cellular proteasome degradation by MG132 completely prevented the IRF-3 degradation mediated by CSFV infection.

### 6.3. Antagonism IFN Induction *via* Inhibiting IRF-3 Activation or Function

As a key transcription factor, IRF-3 is a frequent target of viral proteins to inhibit its phosphorylation, such as HCV NS3/4A protease and PRRSV nsp1β (Table 3) [161,162]. When stimulated with Sendai virus, a potent IFN inducer, HCV NS3/4A serine protease blocks the phosphorylation of IRF-3 as well as IRF-3-mediated promoter activation [162]. Interference of NS3/4A protease function by mutation or an inhibitor restored IRF-3 phosphorylation. For the PRRSV, nsp1β, a self-cleavage product of PRRSV nsp1 during infection, was identified as the antagonist for IRF-3 activation. In 293-TLR3 cells with nsp1β expression, the IRF-3 phosphorylation is reduced to basal level when the cells were stimulated with dsRNA [161]. However, the mechanism of nsp1β blocking the IRF-3 phosphorylation is still unknown. Hepatitis B virus (HBV) is another virus suppressing IRF-3 activation and nuclear translocation [163]. Detailed analysis indicated the HBV polymerase protein can dampen the interaction between TBK1/IKKε and DDX3 [163], while DDX3 has been involved in TBK1/IKKε-mediated IRF-3 activation [164,165]. Furthermore, immediate-early protein 62 of Varicella-Zoster virus (VZV) blocks IRF-3 phosphorylation [166]. However, no interaction of IE62 with TBK1 or IRF-3 was identified, and presence of IE62 did not disrupt the TBK1-IRF-3 complex formation. Moreover, immediate early ICP0 protein from herpes simplex virus (HSV) can recruit activated IRF-3 and CBP/p300 to ICP0 nuclear foci to block IFN-β induction [167].

### 6.4. Antagonism of IFN Induction *via* Virally-Encoded Deubiquitinases

The connection between ubiquitination and activation of the IFN induction pathway has been defined, and cellular and viral deubiquitinases have been demonstrated to play a negative role in IFN induction (Table 3) [168,169,170]. As the cysteine proteases represent a large family of deubiquitinases [171], it implies that viral-encoded cysteine proteases can function as deubiquitinases to inhibit ubiquitination-dependent activation of host IFN signaling. Mounting evidence from virus studies indicates that viral-encoded cysteine proteases indeed possess deubiquitinase function and inhibit host innate immunity, such as arterivirus papain-like protease 2 [172]. The cysteine protease domain located on the N terminus of nsp2 of PRRSV belongs to the ovarian tumor (OTU) protease superfamily. This OTU domain antagonizes the type I IFN induction by interfering with the NF-κB signaling pathway via inhibit the polyubiquitination process of IκBα and prevents IκBα degradation [173,174]. For foot-and-mouth disease virus (FMDV), the leader proteinase (Lpro), a papain-like proteinase, has been demonstrated to process deubiquitinase activity to inhibit IFN induction [175]. Expression of Lpro significantly reduces ubiquitination level of RIG-I, TBK1, TRAF6 and TRAF3, which are key molecules for type I IFN induction [175]. Mutation analysis of Lpro proteolytic core further indicates that the deubiquitinase activity does not rely on the protease activity of Lpro.

Recently, the protease domain of hepatitis E virus was demonstrated to function as a deubiquitinase for RIG-I and TBK1, resulting in inhibition of IFN induction [176]. The deubiquitination of RIG-I and TBK1 can also be observed in HEV-infected hepatoma cell line S10-3 [176]. Furthermore, as an ubiquitin homologue, small ubiquitin-related modifiers (SUMO) cause the sumoylation of IRF-3 and IRF-7, which has been demonstrated as a means of negative regulation of IFN induction by viruses [177]. Upon vesicular stomatitis virus (VSV) infection, IRF-3 and IRF-7 are modified by SUMO1, SUMO2, and SUMO3 [177]. Lys152 of IRF-3 and Lys406 of IRF-7 were identified as the SUMO conjugation sites. Mutation of sumoylation sites in IRF-3/7 led to stronger IFN induction after VSV infection, indicating a negative role for sumoylation in IFN induction.

### 6.5. Antagonism *via* Viral Homologues of Host Molecules

Viruses can encode some homologues of the host TLR and RLR signaling molecules or related molecules to interfere with host signaling, mainly for some DNA viruses with a large genome (Table 3). For example, the products of vaccinia ORFs A46R and A52R, which share sequence similarity with TIR domain, are antagonists of TLR signaling [178,179]. When expressed in mammalian cells, A46R product partially inhibited IL-1-mediated signaling. On the hand, A52R product could mimic the dominant-negative version of MyD88 to block signaling pathways of IL-1, TLR4, and IL-18, but had no effect on MyD88-independent signaling pathways [178].

**Table 3:** Viral antagonism against IFN induction.

Mechanism	Target	Example of viral antagonist [Ref]
Binding to host molecules	RIG-I	NS1 of influenza A [149]
		V of paramyxoviruses [150]
	MDA-5	NS1 of respiratory syncytial virus [151]
	MAVS	γ_1_34.5 of HSV [152]
	TBK1 and iKKε	VP35 of Ebola virus [153]
	IRF3	E6 of Human papillomavirus 16 [155]
Degradation or cleavage of host molecules	RIG-I	3C protease of EMCV [156]
	TRIF MAVS	HAV 3CD [157], coxsackievirus B 3C [158]
	IRF3	Npro of CSFV [160]
Inhibition of IRF3 phosphorylation	IRF3	HCV NS3/4A protease, PRRSV nsp1β, HBV polymerase Varicella-Zoster virus, IE62 protein, HSV ICP0 protein [161,162,163,166,167]
Virally-encoded deubiquitinases	RIG-I, TBK1, TRAF6 and TRAF3	PCP 2 of arterivirus [172], nsp2 of PRRSV [173,174], Lpro of FMDV [175], PCP of HEV [176]
Viral homologues of host molecules	TIR domain, IRF	A46R and A52R of vaccinia [178,179] viral-encoded IRFs of KSHV [180,181]

Kaposi’s sarcoma-associated herpesvirus (KSHV) also antagonizes IFN induction via viral homologues. The KSHV genome has four genes (vIRF-1–4) that showed similarity to cellular IRFs [180,181]. Subsequent studies demonstrated that vIRF-1-3 are able to inhibit transcriptional activation of both type I and type II IFNs, as well as inflammatory signals, ISG and ISRE promoters [181,182]. The vIRF-1 of KSHV was demonstrated to associate with cellular IRF-3 and IRF-7 to inhibit IFN induction [183]. In addition, the vIRF-1 binds to p300/CBP, the transcriptional coactivator for efficient production of IFNβ, to block the IFN induction [183,184]. The vIRF2 was demonstrated to interact with cellular IRF-1, IRF2, IRF8, RelA (p65), and p300 [185]. Although no direct interaction of vIRF2 with IRF-3 was observed [185], vIRF2 was able to enhance caspase-3 mediated degradation of IRF-3, which results in inhibition of the IFNβ expression [186]. For vIRF-3, current data indicates it can associate with IRF-3, IRF5 and IRF-7 to inhibit IFN induction [187,188,189]. However, vIRF-3 only interact with phosphorylated cellular IRF-3 in nucleus, while the interaction between vIRF-3 and IRF-7 is independent of IRF-7 activation [187,188]. Moreover, vIRF-3 does not affect IRF-7 activation and nuclear translocation but disrupt IRF-7 binding with DNA [188].
